# Development of new approach methods for the identification and characterization of endocrine metabolic disruptors—a PARC project

**DOI:** 10.3389/ftox.2023.1212509

**Published:** 2023-06-29

**Authors:** Albert Braeuning, Patrick Balaguer, William Bourguet, Jordi Carreras-Puigvert, Katreece Feiertag, Jorke H. Kamstra, Dries Knapen, Dajana Lichtenstein, Philip Marx-Stoelting, Jonne Rietdijk, Kristin Schubert, Ola Spjuth, Evelyn Stinckens, Kathrin Thedieck, Rik van den Boom, Lucia Vergauwen, Martin von Bergen, Neele Wewer, Daniel Zalko

**Affiliations:** ^1^ Department of Food Safety, German Federal Institute for Risk Assessment, Berlin, Germany; ^2^ IRCM (Institut de Recherche en Cancérologie de Montpellier), Inserm U1194, Université de Montpellier, ICM, Montpellier, France; ^3^ CBS Centre de Biologie Structurale, Université de Montpellier, CNRS, Inserm, Montpellier, France; ^4^ Department of Pharmaceutical Biosciences and Science for Life Laboratory, Uppsala University, Uppsala, Sweden; ^5^ Department of Pesticides Safety, German Federal Institute for Risk Assessment, Berlin, Germany; ^6^ Department of Population Health Sciences, Institute for Risk Assessment Sciences, Faculty of Veterinary Medicine, Utrecht University, Utrecht, Netherlands; ^7^ Zebrafishlab, Veterinary Physiology and Biochemistry, Department of Veterinary Sciences, University of Antwerp, Wilrijk, Belgium; ^8^ Department of Molecular Systems Biology, Helmholtz Centre for Environmental Research (UFZ), Leipzig, Germany; ^9^ Institute of Biochemistry and Center for Molecular Biosciences Innsbruck, University of Innsbruck, Innsbruck, Austria; ^10^ Toxalim (Research Centre in Food Toxicology), Université de Toulouse, Institut National de Recherche Pour L’Agriculture, L’Alimentation et L’Environnement (INARE), Ecole Nationale Vétérinaire de Toulouse (ENVT), INP-Purpan, Université Paul Sabatier (UPS), Toulouse, France

**Keywords:** endocrine metabolic disruption, energy metabolism, obesogens, nuclear receptors, adipocytes, liver

## Abstract

In past times, the analysis of endocrine disrupting properties of chemicals has mainly been focused on (anti-)estrogenic or (anti-)androgenic properties, as well as on aspects of steroidogenesis and the modulation of thyroid signaling. More recently, disruption of energy metabolism and related signaling pathways by exogenous substances, so-called metabolism-disrupting chemicals (MDCs) have come into focus. While general effects such as body and organ weight changes are routinely monitored in animal studies, there is a clear lack of mechanistic test systems to determine and characterize the metabolism-disrupting potential of chemicals. In order to contribute to filling this gap, one of the project within EU-funded Partnership for the Assessment of Risks of Chemicals (PARC) aims at developing novel *in vitro* methods for the detection of endocrine metabolic disruptors. Efforts will comprise projects related to specific signaling pathways, for example, involving mTOR or xenobiotic-sensing nuclear receptors, studies on hepatocytes, adipocytes and pancreatic beta cells covering metabolic and morphological endpoints, as well as metabolism-related zebrafish-based tests as an alternative to classic rodent bioassays. This paper provides an overview of the approaches and methods of these PARC projects and how this will contribute to the improvement of the toxicological toolbox to identify substances with endocrine disrupting properties and to decipher their mechanisms of action.

## 1 Introduction

Endocrine-disrupting chemicals (EDCs) are defined as compounds, which cause an adverse effect in an intact organism, by a toxicological mechanism involving the disturbance of parts of the endocrine system. These characteristics are widely accepted (Solecki et al., 2017) and have been adopted for defining regulatory criteria for endocrine disruptors (European Commission, 2017; 2018). In general, the adverse effects of EDCs can be detected using classic rodent *in vivo* studies, whereas validated mechanistic assays to unequivocally demonstrate an endocrine mechanism of action as the underlying cause of an observed adverse effect *in vivo* are sparse. Current validated methods to identify EDCs are mainly focused on (anti-)estrogenic and (anti-)androgenic mechanisms, as well as on interference with thyroid signaling or steroidogenesis. These endpoints are often referred to as “EATS.”

**FIGURE 1:**
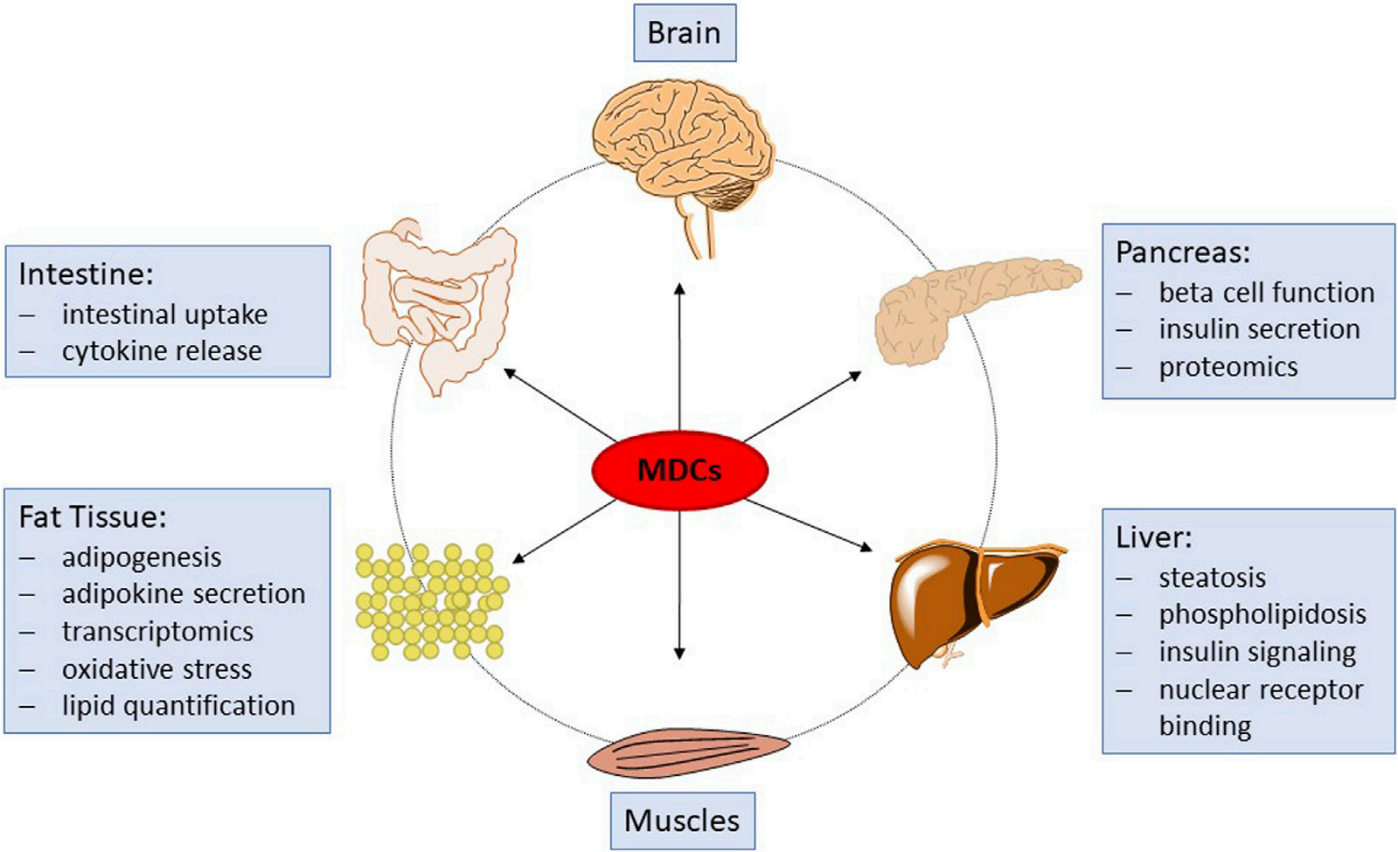
Schematic overview of organs and tissues affected by MDCs, and of the associated endpoints to be investigated within PARC.

Recently, it has been recognized that in addition to EATS modalities, other mechanisms of endocrine disruption may occur. For example, one specific main adverse effect of a number of some EDCs is their capability to induce a lasting disruption of cellular pathways essential for the synthesis or degradation of endogenous compounds and metabolites. These compounds are therefore termed endocrine metabolism-disrupting chemicals (MDCs; also referred to as metabolic EDCs) and may affect, for example, signaling pathways related to cellular energy metabolism (Heindel et al., 2017). MDCs are suspected to contribute to the incidence of obesity and related metabolic disorders, such as type II diabetes and non-alcoholic fatty liver disease, in association with genetic factors, nutrition and lifestyle. Presently, over 50 million people in Europe suffer from metabolic disorders and the potential role of environmental stressors such as MDCs (either man-made or natural chemicals) is being increasingly recognized. Substantial research efforts have recently been made by the European Union to promote the screening, testing, and research on MDCs and their modes of action, for example, within the EURION cluster of EU H2020 projects. However, no suitable *in vivo* or *in vitro* tests fit for regulatory testing of metabolism disrupting effects of chemicals are presently available (OECD, 2018). Since the absence of suitable specific and mechanistic test methods prohibits hazard and risk assessment of chemicals to determine their potential for metabolic disruption, the development of such test methods has been internationally recognized as a high priority (OECD, 2012; Bopp et al., 2017; Manibusan and Touart, 2017; OECD, 2018; Audouze et al., 2020; Browne et al., 2020; Kublbeck et al., 2020; Legler et al., 2020). Screening assays for relevant nuclear receptors, which bind certain MDCs and therefore constitute an early event in the molecular pathogenesis of some metabolic disruption related pathways, are underway (Manibusan and Touart, 2017), but there is also a need for *in vitro* methods that can cover endpoints corresponding to adverse outcomes of metabolic disruption with relevance to human health. Recent endeavors in the development of such assays are part of the ongoing H2020 EU projects EDCMET, OBERON and GOLIATH, addressing important organs, such as liver, pancreas and adipocytes. However, these assays are at different readiness levels from pre-development towards the (pre)- validation stage for some assays. In PARC, we aim to further develop these assays and complement them by new test systems addressing additional endpoints. Taken together, the chosen assays and endpoints will constitute a big step forward in the testing for metabolism-disrupting properties of chemical substances. Figure 1 provides an overview of the different sub-projects and assays and how they fit together to improve future toxicological testing strategies.

## 2 Stable reporter cell lines to determine the potency and efficacy of chemicals on human nuclear receptors

Environmental chemicals including bisphenols (BPA, BPS, BPF), halogenated bisphenols (TBBPA, TCBPA), chemicals used as bisphenol substitutes (BPS-IP, Pergafast 201), and their metabolites will be characterized for their activities at the peroxisome proliferator-activated receptor gamma (PPARγ), the pregnane-X-receptor (PXR) and the constitutive androstane receptor (CAR). To this end, we will use reporter cell lines expressing the ligand binding domain (LBD) of hPPARγ, hPXR or hCAR fused to the yeast GAL4 DNA-binding domain (DBD) (Riu et al., 2011; Grimaldi et al., 2019; Toporova et al., 2020). To characterize their retinoid-X-receptor alpha (RXRα) activities, we will use a retinoic acid receptor beta (RARβ) reporter cell line where both RARβ and RXRα ligands are active (Ren et al., 2023). These reporter cell lines were generated by a two-step transfection procedure. First, stable cell lines expressing the reporter gene were developed (Table 1). Then, the cells were transfected with the different receptor genes. These cell lines stably express chimeric nuclear receptors containing the yeast transactivator GAL4 DBD fused to LBD regions (PPARγ, PXR and CAR reporter cell lines) or in which the DBD was replaced by the estrogen receptor alpha DBD (RARβ reporter cell line) (Table 1). In addition, the HG5LN parental reporter cell line was used as negative control. These nuclear receptor reporter cell lines are powerful tools to characterize the nuclear receptor activity of MDCs in a standardized, high-throughput screening technique. During the project, we will analyze the PPARγ, PXR, CAR or RXRα selectivity and activity of test chemicals. Active MDCs will be compared to reference compounds (Table 1).

**TABLE 1:** Nuclear receptor reporter cell lines and model agonists/antagonists.

Reporter cell line	Reporter gene	Nuclear receptor	Reference agonist	Reference antagonist
HG5LN	GAL4RE_5_-βGlobin-Luc-neomycin			
HG5LN PPARγ	GAL4RE_5_-βGlobin-Luc-neomycin	GAL4-PPARγ-puromycin	Rosiglitazone	T 0070907
HG5LN PXR	GAL4RE_5_-βGlobin-Luc-neomycin	GAL4-PXR-puromycin	SR12813	SPA70
HG5LN CAR	GAL4RE_5_-βGlobin-Luc-neomycin	GAL4-CAR-puromycin	CITCO	PK11195
HELN	ERE-βGlobin-Luc-neomycin			
HELN RARβ (ERα DBD)	ERE-βGlobin-Luc-neomycin	RARβ-puromycin (RXRα endogenous)	CD3254	UV3003

A precise knowledge of the binding mode of MDCs to nuclear receptors is crucial to deeply understand their activity, predict the activity of related compounds and guide the rational development of safer substitutes. To achieve this goal, we will use X-ray crystallography to solve the high-resolution structures of MDCs in complex with their receptor targets. Using this experimental approach, our past work has increased our knowledge of the mechanisms by which various MDCs can substitute for endogenous ligands and alter the normal functions of nuclear receptors, including RXRα (le Maire et al., 2009; Ren et al., 2023), PPARγ (Riu et al., 2011) or PXR (Delfosse et al., 2021). In the PARC project, we will crystallize the purified LBDs of hPXR, hCAR, hPPARγ and hRXRα bound to the most active chemicals identified in our cell-based assays, and elucidate the structures of the corresponding complexes with the aim to establish comprehensive structure-activity relationships.

## 3 Analysis of MDC effects on lipid metabolism in human liver cells

As the central metabolic organ of the body, the liver is highly exposed to MDCs and is one of their main targets. MDCs may interfere with energy, lipid and glucose metabolism pathways, leading to different manifestations of metabolic dysfunction in the liver, for example, to an accumulation of triglycerides or to phospholipidosis, an intracellular lysosomal accumulation of phospholipids with a largely unknown mechanism (Alonso-Magdalena et al., 2015).

Standardized methods to identify and characterize MDCs as well as analysis regarding metabolic functions and endpoints are still lacking. As part of the EU-funded research project EDCMET (www.uef.fi/edcmet; Kublbeck et al., 2020), a test method for measuring hepatic triglyceride accumulation (Lichtenstein et al., 2020) has been optimized in human HepaRG liver cells and is currently on its way towards formal assay validation with the PEPPER platform (Grignard et al., 2022). In order to complement this previous work, it is planned to establish a phospholipidosis assay with differentiated HepaRG cells to screen MDCs for effects on another lipid metabolism-related endpoint. The screening will focus on several substance groups such as pesticides, food additives, perfluorinated compounds, bisphenols, and MDC mixtures. As the liver also plays a major role in glucose metabolism, we want to proceed with a similar approach for establishing a glucose uptake assay in human liver cells. To understand the molecular mechanism leading to phospholipidosis, the mode of action of the most potent MDCs will be analyzed by multi-omics approaches (including transcriptomics and proteomics) to cover key signaling pathways of cellular metabolism. The identification of essential key events by means of bioinformatics data mining approaches will help to close data gaps, to provide mechanistic insights into adverse outcome pathways and to improve the prediction of adverse effects.

Recent research indicates that 3-dimensional cell culture models and the presence of extracellular matrix components improve metabolic activity and physiological behavior (Wang et al., 2021). Moreover, in the context of metabolic disruption, it is useful to not only research isolated cell or organ types, but also to analyze organ-to-organ communication and effects related to an interplay between different tissues. Therefore, an additional aim in PARC is the development of *in vitro* models enabling cells to organize in a 3-dimensional structure which will allow the study of organ interplay in co-cultures of different cell types. Such models will be used to mimic an *in vivo*-like substance uptake and to investigate effects on transport, compound metabolism, and disruption of organ interactions.

## 4 Disruption of pancreatic beta cell function by MDCs

The pancreas is a crucial organ in maintaining metabolism homeostasis through the production of digestion enzymes and hormones by its exocrine and endocrine cells, respectively (Citro and Ott, 2018). Pancreatic beta cells are endocrine cells important for maintaining glucose homoeostasis as they produce, store and secrete the hormone insulin, a key regulator in glucose levels (Seino et al., 2010; Marchetti et al., 2017). An impairment in pancreatic beta cell function is a characteristic of both type 1 and type 2 diabetes mellitus (Eizirik et al., 2020). Studies have shown that MDCs can destroy beta cells, thereby promoting the development of type 1 diabetes mellitus. MDCs also interfere with beta cell signaling and function, resulting in either an increase or decrease in glucose-stimulated insulin secretion. Influence on beta cell division and death have also been attributed to MDCs (Heindel et al., 2017; Legler et al., 2020). Therefore, we want to develop testing methods, which will be able to screen for MDCs’ ability to affect the function of pancreatic beta cells. We have selected the Endoc-βH1 and Endoc-βH5 cell lines as a starting point. Endoc-βH1 have been shown to be a reliable human pancreatic beta cell line capable of secreting insulin as a response to glucose and expresses beta cell marker expression (Tsonkova et al., 2018). We will conduct omics analyses of gene and protein expression to elucidate the specific pathways and processes that are disrupted by MDCs by means of bioinformatics data mining. As MDCs may not only cause effects as a single substance, focus will also be placed on mixture effects. Mixture effects can alter the toxicity of substances, including but not limited to plant protection products (Großkopf et al., 2021; Feiertag et al., 2023) and can be generally grouped into additive, antagonistic, or synergistic effects.

In a whole organism, several organs are responsible for the homoeostasis of glucose, including the intestine, which absorbs glucose, and also the liver, which plays a role in the regulation of glucose (Heindel et al., 2017). Therefore, we plan to create a co-culture with the Endoc-βH1 cells and liver cell lines to simulate the organ to organ communication present in *in vivo* systems.

## 5 Measures for obesogenic effects of MDCs

Epidemiological studies have indicated that MDCs may promote the development of obesity and metabolic syndrome, including type 2 diabetes (Heindel et al., 2017). Despite an increasing number of studies focusing on adipogenesis as an endpoint, a mechanistic understanding of chemically modulated adipogenesis and adipocyte function is still lacking, especially data gaps on a) the suitability of models for adipogenesis and b) the molecular mode of actions. These need to be filled in to develop testing strategies.

Recently, it was demonstrated that BPA and some of its alternatives bind to PPARγ, one master regulator of human adipogenesis. Instead of activating PPARγ, the BPA alternatives studied had an inhibitory effect on human adipogenesis in SGBS cells, which was promoted by inhibiting PPARγ (Schaffert et al., 2021). The exposed adipocytes exhibited an inflammation-like state and dysfunctional properties, e.g., disturbed insulin receptor signaling and altered adipokine release. The BPA substitutes caused similar metabolic dysfunction to BPA and, thus, may promote metabolic dysfunction of adipose tissue, possibly involved in the development of type 2 diabetes. In contrast, in human bone marrow-derived mesenchymal stem cells (hMSCs), BPA alternatives induced adipogenesis (Norgren et al., 2022), stressing the importance to research mechanisms and variable exposure scenarios.

Previously obtained data gained mechanistic insights into how activation of nuclear receptors, like PPARγ or RXRα promote adipogenesis, impair adipose tissue homeostasis and function, potentially resulting in the development of obesity and metabolic syndrome that increases the populations morbidity and mortality (Heindel et al., 2017). However, adverse effects mediated by stressors like bisphenols or other chemicals still could not reveal whether PPARγ is the main molecular initiation event. Disturbed insulin receptor signaling, mTOR signaling, or glucose metabolism were observed to be altered. However, it is still not entirely understood which essential key events are triggered to mediate the adverse effect. In PARC different models for adipogenesis will be employed to fulfil the need for new or improved testing strategies for MDCs (OECD, 2012). During adipogenesis, such compounds can either directly activate or interfere with key processes such as via the activation of PPARγ, or via less researched pathways involving for instance insulin, the mTOR pathway or glucose metabolism. Established adipogenesis models have been used to research MDCs, such as the widely used murine 3T3-L1 model (Kamstra et al., 2014; Kassotis et al., 2021), the human SGBS cell line, or primary human bone marrow-derived human mesenchymal stem cells (hMSCs) (Chamorro-García et al., 2012; Liu et al., 2019; Norgren et al., 2022). The 3T3-L1 model has been tested for robustness in previous initiatives (Christopher et al., 2021) and the hMSC model is being characterized within the EU Horizon 2020 project GOLIATH (Legler et al., 2020). Although these models have been developed using conventional culture methods, research indicates that the use of other culture methods such as spheroids might be more physiologically relevant as these models resemble *in vivo* physiology more closely (Kassotis et al., 2022). Furthermore, present cell cultures use fetal bovine serum containing medium, sometimes up to 15%, which is not in line with the 3R principle.

In PARC, we aim to improve the current testing methods by directly employing the human-relevant SGBS and hMSCs cell models. Using hMSCs we will establish methods for culturing in 3D and in serum free conditions. This model will be fully characterized with transcriptomics analysis, and tested for reproducibility with different known MDCs. A hallmark of unhealthy obesity is inflammation of adipose tissue, often visceral adipose tissue (Kawai et al., 2021). In order to mimic this, we will also combine the model with an intestinal transwell barrier model and investigate the combination of chemical exposures and released cytokines to the health of mature adipocytes. We furthermore aim to assess the effects of BPA alternatives, their potential biotransformation products and mixtures in SGBS adipocytes and on white adipose tissue functionality (secretion of adipokines and adipogenesis) *in vivo*. Using a combined approach with phenotyping, interactome studies to decipher the molecular modes of action (Türkowsky et al., 2019), and multi-omics data to identify relevant signaling pathways affected (Großkopf et al., 2021). Data for AOP elaboration and quantitative *in vitro*-*in vivo* extrapolation will be delivered. Furthermore, detailed analyses of the mTOR signaling pathway, a key regulator of cellular metabolism, will be conducted to decipher the exact modes of action by which MDCs affect this pathway and contribute to the observed adverse outcome.

Our studies will enable the provision of biomarkers to support human biomonitoring, and the work carried out in these projects will initially support hazard assessment and ultimately integrated risk assessment of chemicals, and will include adipogenesis and obesity as further endpoints.

## 6 Zebrafish-based assays to detect metabolism-disrupting chemicals

In this project, different zebrafish life stages are explored as a vertebrate model for metabolic disruption, allowing extrapolation to human health effects. Many endocrine pathways/axes, including receptors, targets, feedback loops, etc. are well conserved across vertebrate taxa. Although there are some differences between zebrafish and humans that are relevant in a metabolic context, such as absence of certain nuclear receptors (RARβ, LXRβ and CAR) (Schaaf, 2017), the zebrafish is considered a valuable model for metabolic disruption in human health (Zang et al., 2018). Several studies have used different life stages of zebrafish as a model to study the impact of chemical exposure on the development of metabolic disorders (Tingaud-Sequeira et al., 2011; Riu et al., 2014; den Broeder et al., 2017; Sun et al., 2019; Martinez et al., 2020; Nakayama et al., 2020; Tian et al., 2021). At present, the zebrafish obesogenic test (ZOT) is the most advanced test method for investigating metabolic disruption in the zebrafish model (Tingaud-Sequeira et al., 2011). In this test, larvae are raised on control diet until they reach a length between 7.5 and 9 mm (3–4 weeks post fertilization). The experimental treatment consists of 1 day of feeding with a high-fat diet, followed by 1 day of starvation, and 1 day of aquatic exposure to the compound of interest. The endpoint of this test is a whole-body adiposity measurement using Nile Red staining. Among others, this test has been shown to be responsive to known PPAR-modulating compounds (Riu et al., 2014). In general, more insight into the mechanisms and effects of metabolic disruptors can be gained when different life stages, exposure scenarios and endpoints are used.

We will focus specifically on zebrafish early-life stages in order to explore different potential strategies to assess mechanisms and effects of metabolic disruptors. We will investigate responses to metabolic disruption in two different life stages, applying different exposure strategies and measuring a set of relevant endpoints. First, zebrafish embryos will be used. They are not protected under the current EU legislation on the use of laboratory animals until the age of free-feeding, which is at 5 days post fertilization when kept at 28°C (European Commission, 2012). On one hand, this is an advantage in terms of reducing animal testing and on the other hand the absence of exogenous feeding prohibits dietary intervention. There is, however, an added layer of complexity related to the fact that the liver and digestive system develop during the exposure period. Secondly, larval assays like the ZOT allow for dietary interventions, including diets with altered composition as well as for dietary exposure to compounds. We aim to expand upon the ZOT’s basic principles in terms of life stage used, exposure duration, and endpoint complexity. Custom fish feeds such as a western diet (high fat, high cholesterol, high carbohydrate content including fructose) are under development based on previous experiences (Gabriels et al., 2019). Endpoints with relevance to human health will be explored, such as the condition factor (corresponding to body mass index), whole organism lipid staining and quantification, oxidative stress and transcription of genes related to endocrine disruption and energy metabolism. A case study with exposure to bisphenol A and some of its alternatives is envisioned, based on PARC priority areas.

An embryonic zebrafish metabolic disruption test would be very valuable since it would combine the advantages of a whole organism assay with the facts that it is considered an alternative to animal testing and it is a new approach methodology (NAM). Larval feeding trials such as the ZOT are animal tests. However, as also suggested by Tingaud-Sequeira (2011) in relation to the ZOT, zebrafish early-life stage metabolic disruption testing could be an intermediate step in a tiered approach between *in vitro* assays and rodent tests. As such, this approach would aid in reducing mammalian testing.

## 7 Summary and outlook

As detailed above, PARC will substantially contribute to the field of metabolic disruption by chemicals. This will especially comprise the collection of data for important groups of endocrine active compounds, e.g., bisphenols, as well as the development and implementation of new *in vitro* or non-rodent *in vivo* test methods to identify and characterize MDCs. Moreover, the metabolic disruption-related projects in PARC will elucidate molecular mechanisms of toxicity and thus contribute to the improvement of AOP networks.

## Data Availability

The original contributions presented in the study are included in the article/supplementary material, further inquiries can be directed to the corresponding author.
