# Corrigendum: Development of new approach methods for the identification and characterization of endocrine metabolic disruptors—a PARC project

**DOI:** 10.3389/ftox.2024.1394396

**Published:** 2024-03-25

**Authors:** Albert Braeuning, Patrick Balaguer, William Bourguet, Jordi Carreras-Puigvert, Katreece Feiertag, Jorke H. Kamstra, Dries Knapen, Dajana Lichtenstein, Philip Marx-Stoelting, Jonne Rietdijk, Kristin Schubert, Ola Spjuth, Evelyn Stinckens, Kathrin Thedieck, Rik van den Boom, Lucia Vergauwen, Martin von Bergen, Neele Wewer, Daniel Zalko

**Affiliations:** ^1^ Department of Food Safety, German Federal Institute for Risk Assessment, Berlin, Germany; ^2^ IRCM (Institut de Recherche en Cancérologie de Montpellier), Inserm U1194, Université de Montpellier, ICM, Montpellier, France; ^3^ CBS Centre de Biologie Structurale, Université de Montpellier, CNRS, Inserm, Montpellier, France; ^4^ Department of Pharmaceutical Biosciences and Science for Life Laboratory, Uppsala University, Uppsala, Sweden; ^5^ Department of Pesticides Safety, German Federal Institute for Risk Assessment, Berlin, Germany; ^6^ Department of Population Health Sciences, Institute for Risk Assessment Sciences, Faculty of Veterinary Medicine, Utrecht University, Utrecht, Netherlands; ^7^ Zebrafishlab, Veterinary Physiology and Biochemistry, Department of Veterinary Sciences, University of Antwerp, Wilrijk, Belgium; ^8^ Department of Molecular Systems Biology, Helmholtz Centre for Environmental Research (UFZ), Leipzig, Germany; ^9^ Institute of Biochemistry and Center for Molecular Biosciences Innsbruck, University of Innsbruck, Innsbruck, Austria; ^10^ Toxalim (Research Centre in Food Toxicology), Université de Toulouse, Institut National de Recherche Pour L’Agriculture, L’Alimentation et L’Environnement (INARE), Ecole Nationale Vétérinaire de Toulouse (ENVT), INP-Purpan, Université Paul Sabatier (UPS), Toulouse, France

**Keywords:** endocrine metabolic disruption, energy metabolism, obesogens, nuclear receptors, adipocytes, liver

In the published article, there was an error. In **Section 5** an erroneous entry was introduced, indicating an opposite effect of what was shown in the reference referring to that entry.

A correction has been made to ‘*5 Measures for obesogenic effects of MDCs*’, *paragraph 2*. This sentence previously started:

“In contrast, in human bone marrow-derived mesenchymal stem cells (hMSCs), BPA alternatives attenuated adipogenesis (**Norgren et al., 2022**), stressing the importance to research mechanisms and variable exposure scenarios.”

The corrected sentence appears below:

“In contrast, in human bone marrow-derived mesenchymal stem cells (hMSCs), BPA alternatives induced adipogenesis (**Norgren et al., 2022**), stressing the importance to research mechanisms and variable exposure scenarios.”

The authors apologize for this error and state that this does not change the scientific conclusions of the article in any way. The original article has been updated.

